# Supplementary material for: Inhibition of Adult Neurogenesis in Male Mice after Repeated Exposure to Paracetamol Overdose
Source: Int J Mol Sci. 2024 Feb 6;25(4):1964. doi: 10.3390/ijms25041964 (PMC10888347; doi:10.3390/ijms25041964)

Supplementary Materials:

Figure S1: Representative micrographs of occludin expression in the hippocampus of control or repeated administration of acetaminophen (APAP) at a dose of 750 mg/kg/day treated mice.

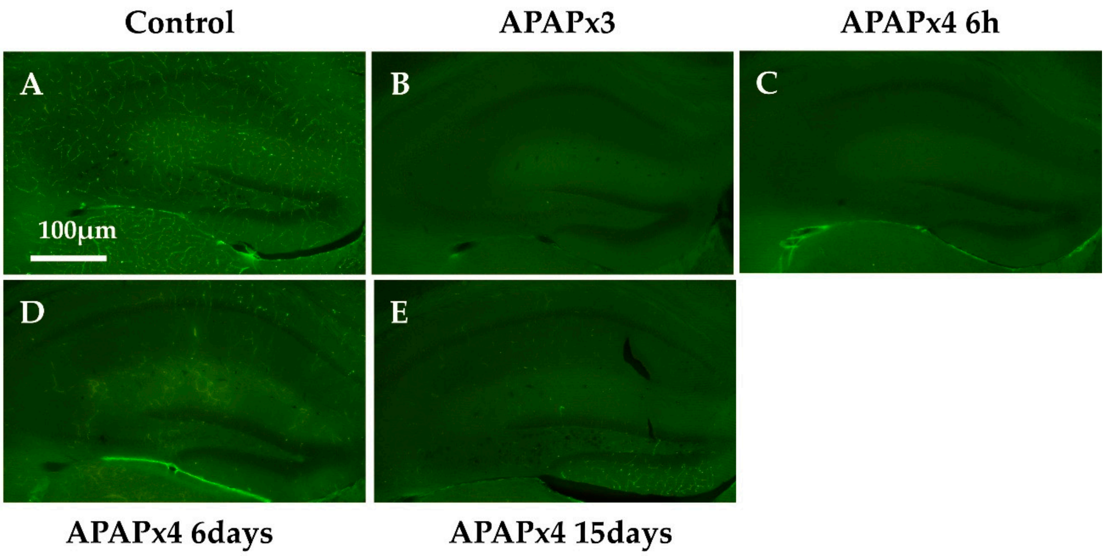

Figure S2: The reduced glutathione (GSH)/oxidized glutathione (GSSG) ratio in the hypothalamus and plasma of control or three repeated administration of acetaminophen (APAPx3) at a dose of 750 mg/kg treated mice.

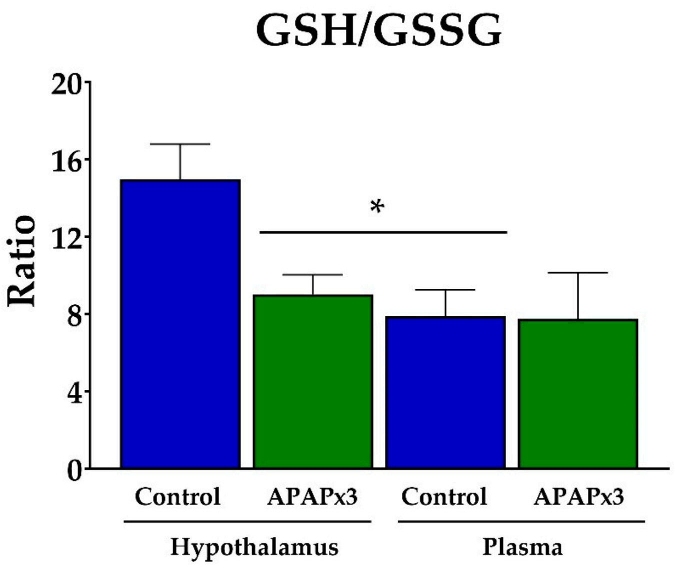

Supplement: Supplementary file 1 [file ijms-25-01964-s001.zip › ijms-2813760-supplementary.pdf]
